# The Complex Fine-Tuning of K^+^ Fluxes in Plants in Relation to Osmotic and Ionic Abiotic Stresses

**DOI:** 10.3390/ijms20030715

**Published:** 2019-02-07

**Authors:** Isabelle Chérel, Isabelle Gaillard

**Affiliations:** BPMP, Univ Montpellier, CNRS, INRA, SupAgro, Montpellier, France; isabelle.gaillard@inra.fr

**Keywords:** plants, abiotic stress, abscisic acid, potassium transport regulation

## Abstract

As the main cation in plant cells, potassium plays an essential role in adaptive responses, especially through its involvement in osmotic pressure and membrane potential adjustments. K^+^ homeostasis must, therefore, be finely controlled. As a result of different abiotic stresses, especially those resulting from global warming, K^+^ fluxes and plant distribution of this ion are disturbed. The hormone abscisic acid (ABA) is a key player in responses to these climate stresses. It triggers signaling cascades that ultimately lead to modulation of the activities of K^+^ channels and transporters. After a brief overview of transcriptional changes induced by abiotic stresses, this review deals with the post-translational molecular mechanisms in different plant organs, in *Arabidopsis* and species of agronomical interest, triggering changes in K^+^ uptake from the soil, K^+^ transport and accumulation throughout the plant, and stomatal regulation. These modifications involve phosphorylation/dephosphorylation mechanisms, modifications of targeting, and interactions with regulatory partner proteins. Interestingly, many signaling pathways are common to K^+^ and Cl^−^/NO3^−^ counter-ion transport systems. These cross-talks are also addressed.

## 1. Introduction

Global warming is impacting crop yield and quality. Increases in atmospheric CO_2_ and light intensity, erratic rainfalls, periods of drought stress, and heat waves are the climatic components most affecting terrestrial plants. Also, an increase in irrigation use leads to soil salinization. Drought and heat stresses, especially when combined, are responsible for significant losses in agriculture [1,2,3]. Different strategies can be applied to lessen their effects, relying on modifications of agricultural practices, isolation of the best genotypes, and transgenesis [3]. This requires knowledge of the mechanisms that govern plant adaptation to these adverse conditions. The different forms of signal transduction in response to drought, salt, and heat stresses from membrane sensors to the terminal cell responses in plant cells were documented [4]. Abscisic acid (ABA) plays an essential role in responses to drought and salt/osmotic stresses [4,5,6,7] and data obtained with the plant model *Arabidopsis thaliana* also indicate that ABA plays a role in the adaptation to high temperatures [6,8,9].

In plants, the synthesis of the phytohormone ABA, which probably occurs mainly in vascular tissues [10], is induced by abiotic stresses [5]. ABA is then transported to target tissues via both xylem and phloem, which allows transport in both directions between roots and shoots [10,11]. Also, drought stress leads to a transient pH increase in xylem sap and apoplast, favoring the dissociation of ABA to its ionic form that would be accumulated in the apoplast of all plant tissues [12]. This is thought to function as a root-to-shoot signal leading to leaf transpiration decrease. It is interesting to note that the transport of ABA to different sites leads to organ-specific and cell-type-specific responses. Finally, coordinated fluxes of K^+^ and anions across cellular membranes typically occur at the end of the biological responses to environmental stimuli.

Modulation of K^+^ fluxes is a well-known response to ABA drought and salt stress. K^+^ is the main cation in plant cells, essential for plant growth and adaptation to the environment [13,14]. Due to its abundance in plant cells, it is involved in neutralization of negative charges, pH homeostasis, and control of the electrical membrane potential [15]. Its concentration in the cytosol is maintained around 100–200 mM, and, in this compartment, it cannot be replaced by another cation such as Na^+^ because the latter affects the water H-bonding at the protein surface more than K^+^ [16]. In contrast, vacuolar K^+^, which is involved in cell turgor, can to some extent be replaced by other osmotica [17,18]. As a consequence of its function in cell turgor, K^+^ is involved in nasties, including stomatal movements [19]. K^+^ also displays biochemical properties. It is involved in the direct or indirect activation of enzymes involved in metabolic processes, such as carbon metabolism and protein synthesis [19]. In K-deficient plants, photosynthesis is also impaired. This is due to the prominent role of K^+^ in mesophyll CO_2_ conductance, chloroplast organization, improvement of Rubisco activity, and translocation of photoassimilates in the phloem [20]. Under high light, K^+^ limits reactive oxygen species (ROS) production that leads to photoinhibition [20]. Maintaining K^+^ homeostasis even under stressful conditions is, therefore, a necessity for plant growth and adaptation [21]. Drought stress impairs K^+^ uptake and, thus, exacerbates the effects of K^+^ deficiency [21]. It also decreases K^+^ translocation to the xylem [22], thus maintaining root growth [23], and triggers K^+^-mediated stomatal closure for preventing dehydration. Salinity also results in root and shoot decline in K^+^, salt tolerance being correlated to the ability to save K^+^, and to the cytosolic K^+^/Na^+^ ratio [21,24]. Indeed, it was documented that K^+^ homeostasis is particularly critical for plant tolerance to drought and salt stresses [21,23] and high light intensity [25].

K^+^ transport systems are targets of signaling pathways, leading to environmental responses such as K^+^ uptake, translocation, and stomatal movements. Multiple channels and transporters were identified in plants [26]. In this review, we focus on regulations of K^+^ transport systems which drive K^+^ fluxes as essential end responses to different abiotic stresses and ABA signaling cascades, and on their relationships with those regarding mineral anion (Cl^−^, NO3^−^) transports.

## 2. K^+^ Transport in Plants

### 2.1. Structure and Function of K^+^ Transport Systems

#### 2.1.1. K^+^-Selective Channels

**The Shaker channels:** K^+^ channels of the Shaker family mediate the major K^+^ fluxes at the plasma membrane [27], and are the best characterized. They are tetramers of four subunits arranged around a central pore, selective for K^+^ ions. Each subunit is made up of a short cytosolic N-terminal sequence, a membrane part containing the pore-forming domain with the K^+^ selectivity filter, and a large C-terminal intracytoplasmic part including a cyclic-nucleotide-binding domain, an ankyrin repeat protein–protein interaction domain, and a conserved hydrophobic and acidic (KHA) C-terminal end. In *Arabidopsis thaliana*, Shaker channels are encoded by a family of nine genes, which are grouped into five subfamilies (for reviews, see References [15,27]). The preferential assembly of these subunits as heterotetrameric structures enables a multiplicity of combinations increasing functional diversity [28,29,30]. Shaker channels are voltage-gated. Depending on the subfamily, Shaker subunits form inwardly rectifying (Kin) or outwardly rectifying (Kout) channels (activated by membrane hyperpolarization or depolarization, respectively). In *Arabidopsis*, AKT1, AKT5, AKT6 (*Arabidopsis* K^+^ transporters 1, 5 and 6), KAT1, KAT2 (K^+^ *Arabidopsis* transporters 1 and 2), and AtKC1 (*Arabidopsis thaliana* K^+^ channel 1) subunits assemble as Kin channels, whereas SKOR (stelar K^+^ outward rectifyer) and GORK (guard cell outward rectifying channel) subunits form Kout channels [27]. No assembly could be detected between Kin and Kout channel subunits [31]. A notable exception to the strict voltage dependence is AKT2, a “weakly rectifying” channel, that switches from an inwardly rectifying to a non-rectifying state [31]. At the macroscopic level, this results in the superposition of two current components; a “leak-like” component of AKT2 channels open in the whole range of physiological membrane potentials, and an inward rectifying component that closes upon depolarization. Furthermore, AtKC1 is thought to be a regulatory subunit. Indeed, AtKC1 subunits do not form functional homotetrameric channels on their own but they assemble with inward-channel-forming subunits to give rise to heteromeric channels that activate at much more hyperpolarized potentials compared to homotetramers [32,33,34]. Patch-clamp analyses on root hair protoplasts reveal that inward K^+^ currents are likely to correspond to AKT1/AtKC1 heteromers rather than AKT1 homotetramers [35]. Shaker channels are present in all plant organs [36]. They are involved in main functions such as uptake of K^+^ from the soil (even from K^+^ solutions as dilute as 10 µM) and its secretion into the xylem, transport of solutes in the phloem, stomatal movements, and cell elongation (Table 1). Members of the different Shaker gene subfamilies were identified in all studied plant species such as rice, tomato, maize, poplar, grapevine, etc. [27].

**TPKs (Two-pore K^+^ channels)** constitute the other group of K^+^-selective channels. This family comprises six members in *Arabidopsis*. Except for KCO3 (Ca^2+^ activated outward rectifying K^+^ channel 3), which could be an inactive channel, they are dimers of two-pore domain-subunits, composed of four transmembrane segments with two pore domains including the K^+^-selectivity motif GYGD. Of these, only TPK4 (two-pore K^+^ transporter 1) is targeted to the plasma membrane [15,37], with the four other TPKs (TPK1, 2, 3, and 5) located on endocellular membranes. The roles of TPK channels are much less well understood than those of Shakers. TPK4, which is blocked by extracellular Ca^2+^ and cytoplasmic acidification, is an open rectifier channel. This channel is mainly expressed in pollen and is thought to contribute to the K^+^ conductance of the pollen tube plasma membrane [38]. The subunits of TPK1, 2, 3 and 5 channels also contain binding sites for 14-3-3 proteins in the cytosolic N-terminal part and two Ca^2+^-binding EF hands in the cytosolic C-terminal part. TPK3 is expressed in the stromal lamellae of thylakoid membranes where it controls the transmembrane ∆pH and, thus, the light-induced proton motive force [39]. The tonoplast TPK1 channel is ubiquitously expressed. It mediates the VK voltage-independent, K^+^-selective currents observed in guard cells and mesophyll cells [40,41]. In guard cells, it is important for vacuolar K^+^ release during stomatal closure [40,41]. This channel is involved in intracellular K^+^ homeostasis [40]. The biophysical features and physiological functions of TPK 2 and 5 remain unknown.

#### 2.1.2. K^+^-Selective KUP/HAK/KT Transporters (K^+^ Uptake Permease, High Affinity K^+^, K^+^ Transporters)

This family comprises 13 members in *Arabidopsis*. The most popular are AtHAK5 from *Arabidopsis* and its homologs in other species, which mediate high-affinity K^+^ uptake in root from very dilute soil solutions, below 10 µM (reviewed by References [83,84,85]). The energy for K^+^ uptake would be provided by co-transport with protons [83]. KUP transporters display multiple functions in plants. In roots, AtKUP1 is a dual-affinity transporter mediating K^+^ uptake [86], whereas KUP 2, 4, and 6 negatively impact K^+^ (Rb^+^) uptake, and are supposed to mediate K^+^ efflux [68]. KUPs are found at the plasma membrane and in endomembranes, and, in addition to K^+^ uptake from the soil, they are also involved in K^+^ homeostasis, long-distance K^+^ transport, cell elongation, response to osmotic stress, and other unexpected functions such as adenylate cyclase activity and regulation of auxin transport [84].

#### 2.1.3. Cation Transporters

The HKT transporter family is divided into two subfamilies, “Na^+^-selective” and “K^+^-permeable”, which can also transport Na^+^. The latter subfamily, which seems to be specific to monocots, is characterized by the presence of a conserved glycine in the selectivity filter [27]. HKT transporters mediate Na^+^ or K^+^ influx, or Na^+^/K^+^ co-transport [27,83]. NHX (Na^+^/H^+^ exchangers) and CHX (cation/H^+^ exchangers) are cation transporters found on endomembranes including the tonoplast, and CHX transporters are also present at the plasma membrane. Some of them are involved in the control of K^+^ homeostasis [83,87]. These families, poorly characterized with regard to post-translational regulatory networks, are not included in the present review.

### 2.2. Role of Potassium Transport Systems in Adaptation to Stress

Genes encoding membrane transporters and their transcriptional regulations by climate-related stresses (drought, osmotic stress, salt, heat) are presented in Table 1.

#### 2.2.1. K^+^ Uptake and Transfer to the Xylem in Roots

As reported above, maintaining sufficient K^+^ uptake is critical to reduce the effects of drought stress. K^+^ is taken up from a soil solution that is highly variable in composition, with K^+^ concentration that can range from a few µM to several mM. In *Arabidopsis*, below 10 µM K^+^ and in the absence of NH4^+^, only HAK5 is able to mediate K^+^ uptake, whereas AKT1 intervenes at higher, but still low, K^+^ concentrations, due to the high hyperpolarization of root cell membranes in K^+^-deficient soils [88,89]. Salinity strongly represses *HAK5* expression in roots [67]. However, HAK5 is critically required for plant growth under low-K and saline conditions, because Na^+^ absorption results in depolarization of membranes of external root cells, which promotes an unusual K^+^ efflux via AKT1 [67]. In these experiments, the involvement of the outwardly rectifying GORK channel, also known to mediate K^+^ efflux under salt stress [90] (see below), was not addressed. Closest homologs of HAK5 belong to clade 1a of the KUP family [66]. They are involved (or thought to be involved) in K^+^ uptake from the soil at low K^+^ concentrations [27]. Like *HAK5*, homologous genes in other plant species (rice, barley, pepper, tomato, *Thellungiella halophila*) are highly upregulated by K^+^ shortage; however, for at least three of them, this upregulation is suppressed by Na^+^ [27]. Although AKT1 and HAK5 seem to be the main players for K^+^ uptake from the soil even in the low-K^+^ range [88], AtKUP7 in *Arabidopsis* (clade V [66]) was recently found to also contribute to K^+^ uptake under low-K^+^ conditions [91]. In parallel, the AtKC1 subunit, which is expressed in the root cortex, epidermis, and root hairs [44], and interacts with AKT1 to form a functional heterotetrameric channel, prevents K^+^ loss through AKT1 under low-K^+^ conditions [33]. Neither AKT1, HAK5, AtKUP7, nor AtKC1 appear to display strong and durable up- or downregulations at the transcriptional level in response to water stress, heat, or ABA in roots [44,45]. In rice, OsAKT1 also contributes to a significant extent to K^+^ uptake in roots, in the absence of regulation by an AtKC1-like subunit [46]. It improves resistance to drought stress triggered by polyethylene glycol (PEG) or reduction of soil watering, by increasing K^+^ content in roots. Conversely, it has no effect on resistance to salinity [47].

The Kout channel GORK is also expressed in roots [65]. In the case of salt stress, loss of K^+^ must be avoided to maintain the K^+^/Na^+^ ratio. However, Na^+^ entry depolarizes the plasma membrane and evokes massive loss of K^+^ mainly through GORK [90,92,93]. Non-selective cation channels [93] are also significant players of K^+^ efflux. In pea, they assume this function in place of GORK [94]. In *Arabidopsis*, KUP6 and KUP8 (clade II [66]) are also thought to mediate K^+^ efflux from roots [68]. *KUP8* is downregulated under salt stress, but not the *GORK* gene [56]. Conversely, *GORK* is upregulated by heat stress in roots [56]. The reduction of net K^+^ influx observed in olive tree roots exposed to heat [95] might, thus, be attributed to an increase in K^+^ efflux, but this has to be confirmed. Although the role of GORK-mediated K^+^ efflux is much less understood than in guard cells, it is thought to be involved in repolarization of the root-hair plasma membrane, for instance, after elicitor-induced depolarization [96] or in response to different stresses [93]. GORK is activated by ROS (more specifically hydroxide radicals) that are generated following different stresses including salt stress [90]. Depending on stress intensity, GORK-mediated K^+^ efflux could either trigger programmed cell death or prevent activation of anabolic enzymes by K^+^, thus releasing energy for adaptation to stress and reparation [93]. Repolarization of the plasma membrane could have a role in action potentials and propagation of stress and hormone electrical signaling [93].

Once taken up by epidermal and cortical cells at the root periphery, K^+^ is transported radially (via an apoplastic or symplastic pathway) and then delivered to the xylem. KUP6 and KUP8 are expressed in the pericycle, and they enhance ABA response to inhibit lateral root formation [68]. The SKOR channel drives about 50% of K^+^ transported in the xylem sap to the shoot [63]. The *SKOR* gene transcript is considerably reduced in response to ABA treatment [63], in accordance with the previously observed strong decrease of outward K^+^ currents in the maize stele [22]. This would ensure that roots can keep growing under stress, and is also expected to provide a signal for drought stress to the shoots [97], since the decrease in K^+^ content in the xylem sap is accompanied by an increase in Ca^2+^ [63] that serves as an intermediate for signaling cascades. Alongside its role in K^+^ uptake from roots, KUP7 was identified as a transporter allowing K^+^ efflux to the xylem especially under K^+^-deficient conditions (accounting for about one-third of xylem K^+^ [91]). Systematic studies on whole plants do not reveal transcriptional regulation by drought, ABA, or heat for the *KUP7* gene [56]. KUP7 could, therefore, ensure a minimal transfer to the xylem when *SKOR* expression is repressed by ABA. In rice, at least two transporters of the KUP family (OsHAK1 and OsHAK5), belonging to the same clade Ia as HAK5, mediate not only K^+^ uptake from the soil but also translocation to the xylem, possibly (at least for OsHAK5) by allowing K^+^ loading of xylem parenchyma cells prior to K^+^ efflux via SKOR-like channels [85,98]. A recent report [69] highlighted the role of OsHAK1 from rice in drought stress tolerance. For its part, OsHAK5 promotes salt resistance by increasing the K^+^/Na^+^ ratio [70]. OsHAK21 (clade Ia, [66]), mainly expressed in xylem parenchyma cells, is involved in K^+^ uptake and control of K^+^ homeostasis under salt stress [71].

#### 2.2.2. K^+^ Loading and Unloading in the Phloem

Due to the difficulty to access phloem sap and phloem membranes, little is known on the behaviors of phloem K^+^ transport systems during adaptation to stress. The first transport system identified in the phloem was the Shaker channel AKT2 (also referred to as AKT3) from *Arabidopsis* [54,99]. *KAT1* and *KCO6* (*AtTPK3*) transcripts were also detected in isolated phloem companion cell protoplasts [100]. The *KAT2* [60], *OsHAK1* [98], and *OsHAK5* [70] promoters are active (in fusion with the *GUS* reporter gene) in phloem tissues. Due to its unique gating properties, AKT2 was extensively studied with the aim of understanding its role in the phloem transport of solutes. The AKT2 channel from *Arabidopsis* and its counterparts in other species substantially contribute to the phloem K^+^ conductance in both sieve elements and companion cells, where they have a major role in phloem K^+^ loading and unloading [52,54,99,100,101,102,103,104]. Surprisingly, the phloem sap of the *akt2* mutant plant is not depleted in K^+^. Instead, it contains only half the wild-type content of sucrose [52]. This is expected to result from the effect of a “leak” channel on membrane polarization, preventing membrane depolarization during sucrose uptake by the sucrose/proton symporter. The *AKT2* transcript is increased in leaves in response to ABA, thus possibly favoring K^+^ recirculation to the root under drought stress [54].

#### 2.2.3. K^+^ Fluxes in Guard Cells

Most of the literature on drought stress adaptation deals with stomatal regulation. In shoots, water-saving is tightly controlled by ion-driven guard-cell movements. Due to their importance in plant hydric status preservation and their accessibility to (electro)physiological studies, guard cells became a model for the regulation of ion (notably K^+^) fluxes in response to drought stress and ABA. Also, the molecular mechanisms of light-induced stomatal opening and closure were extensively studied (reviewed by References [105,106,107,108,109]). Comparatively to drought stress-induced stomatal closing and light-induced stomatal opening, little is known about the effect of high temperatures on stomatal movements. Under high temperatures and drought conditions, guard cells have to face the dilemma of saving water while controlling leaf temperature through transpiration [110].

ABA perception by PYR/PYL/RCAR (pyrabactin resistance, pyrabactin resistance-like, regulatory component of ABA receptor) ABA receptors triggers an increase in cytoplasmic calcium (due to release from internal stores and Ca^2+^ uptake, regulated by ROS and nitric oxide [42,111,112,113]) leading to activation of anion channels (slow- and quick-activating SLAC1 (slow anion channel 1), SLAH3 (SLAC1 homolog), and QUAC1 (quick anion channel 1)) [114] that depolarize the plasma membrane. The plasma membrane depolarization, in turn, triggers the opening of the K^+^ voltage-gated GORK channel that mediates K^+^ efflux [64,115]. This release of ions drives water out of guard cells, which causes guard-cell deflating and stomatal closure [108,109]. In addition to GORK, KUP6 and KUP8 are also involved in the control of stomatal closure. *KUP6* is highly responsive to osmotic stress, especially in shoots [56,68]. KUP6 over-expressors exhibit a clear drought resistance phenotype, thus highlighting the importance of the *KUP6* gene in stress adaptation, and close their stomata more efficiently than wild-type plants in the presence of ABA [68]. Shaker genes *KAT1*/*KAT2*, *AKT1*, *GORK*, and, to a much lesser extent. *AKT2* and *AtKC1* are expressed in guard-cell protoplasts [55,96,116]. *KAT1* and *KAT2* encode inward-rectifying-forming subunits that, after tetramerization, form the KAT1 and KAT2 channels, which are the key actors of stomatal opening. Stomatal opening mechanism is an inversion of stomatal closure in terms of osmolyte accumulation and direction of fluxes. KAT1 and KAT2 mediate K^+^ influx that drives water influx and opens stomata [61]. Upon ABA exposure, *KAT1* and *KAT2* genes are downregulated at the transcriptional level, as expected for genes involved in stomatal opening [55,117]. The *GORK* gene transcription, which is clearly upregulated by ABA in most tissues [65] was unexpectedly found insensitive (or weakly sensitive) to ABA in guard-cell protoplasts [65,73], thus revealing a specific type of regulation for these cells. AKT1, previously found to be essential for root K^+^ uptake [118], was only recently identified as a player of stomatal movements. In the *akt1* loss-of-function mutant, the stomatal conductance is lower under drought stress and ABA has a much stronger effect on stomatal closing [43].

Although most data rely on plasma membrane channel regulation, it should be emphasized that vacuoles, which serve as solute reservoirs, play a significant role in stomatal movements. The voltage-independent, K^+^-selective TPK1 channel from *Arabidopsis* is essential for the control of K^+^ homeostasis and vacuolar K^+^ release during ABA-mediated stomatal closure [41,119].

#### 2.2.4. K^+^ Fluxes in Reproductive Organs

Pollen germination on the stigma requires cell elongation maintained by K^+^ uptake through the *Arabidopsis* SPIK channel [120]. Data are also emerging regarding the contribution of different K^+^ transport systems to the mechanisms of response to stress and their consequences on fruit quality. Most of them were obtained on grape berry, considered as a model for fleshy fruits [50]. Whereas K^+^ is often limiting in plants, especially under hot and dry climates, it is sometimes accumulated in excess. In grape berries, potassium accumulates after the véraison stage that marks the beginning of maturation with change in berry color and firmness, dramatic increase in sugar content, loss of acidity, and development of aromas. This K^+^ accumulation increased during the past decades and this was attributed to global warming, since high temperatures favor K^+^ accumulation in berries [121]. Although K^+^ seems to be beneficial for enhancing berry resistance to drought [122], it can be deleterious for wine quality. Indeed, due to the neutralization of organic acids by K^+^ ions, high amounts of K^+^ in the must are associated with high pH [122], resulting in wines with loss of aromas and poor aging potential [122,123]. After véraison, due to the loss of xylem functionality for solute transport [124], K^+^ enters in the berry mainly via the phloem. The weakly rectifying Shaker channel VvK3.1 which belongs to the AKT2 channel phylogenetic branch, would be involved in the massive K^+^ fluxes from the phloem cell cytosol to the berry apoplast during berry K^+^ loading [59]. Once delivered to the apoplastic space, K^+^ is taken up by flesh and skin cells [122] to be stored in vacuoles. Several transport systems related to these functions were identified. Two Kup transporters are involved in K^+^ uptake into the skin cells at pre-véraison stages [125]. VvK1.1, the ortholog of AKT1, is expressed in the phloem and in seeds and is strongly upregulated by water stress and ABA in berries not only before but also after véraison [50]. However, its expression level in berries is far below that of the other AKT1-type gene, VvK1.2, which bursts after véraison and is highly induced by water stress [51]. In light of its expression pattern, VvK1.2 is expected to mediate K^+^ retrieval by perivascular and flesh cells from the apoplast.

## 3. Post-Translational Regulations Linked to Osmotic, Salt, or Water Stress and Heat

### 3.1. Regulation by Kinases, Phosphatases, and Associated Proteins

Three main kinase families share the regulation of K^+^ membrane transport system activity: the SnRK2 (SNF1-related protein kinase 2) family, represented notably by OST1/SnRK2.6/SnRK2E [126], the SnRK3 family (SNF1-related protein kinase 3 or CIPK; calcineurin B-like-interacting protein kinase [127]), and the CDPK/CPK family (calcium-dependent protein kinase) [128] (Table 2). All are involved in signaling pathways related to different stresses such as K^+^ deficiency, drought, osmotic, and salt stresses [14,129,130]. The *Arabidopsis* genome encodes 10 SnRK2s, of which OST1/SnRK2.6 is a positive regulator of ABA-dependent stomatal movements [126]. The SnRK2 family is involved in ABA-dependent, Ca^2+^-independent signaling, whereas CIPK and most CPK kinases depend on calcium for their activity [127,131]. CIPKs are activated by interaction with calcineurin B-like (CBL) proteins that bind calcium, which is released in cells in response to different stimuli. They are composed of a kinase domain, an auto-inhibitory (NAF/FISL) domain, and a phosphatase-binding (protein phosphatase interaction, PPI) domain. The calcium-dependent CBL interaction with the NAF domain of the CIPK suppresses the auto-inhibition [132]. CBLs also require to be phosphorylated by their CIPKs to be fully active [133]. There are 26 CIPKs and 10 CBLs in *Arabidopsis thaliana*, which are combined to regulate ion homeostasis and response to ABA in a highly specific manner [134]. In contrast to CIPKs, CPKs (34 members in *A. thaliana*) contain an intrinsic C-terminal calmodulin-like domain comprising EF-hand motives that directly bind calcium. When calcium levels are low, kinases are auto-inhibited, but calcium elevation triggers binding to EF-hands and conformational changes that relieve the auto-inhibition [131]. Calcium sensitivity appears to be highly variable in the CPK family, since some CPKs such as CPK13 and CPK23 seem to be insensitive or much less sensitive to calcium than the others [131]. It is important to notice that members of these SnRK2, CIPK, and CPK families interact with clade A protein phosphatases 2C, known to be involved in ABA signaling [78,135,136,137,138,139,140,141]. These phosphatases are trapped and inactivated by PYR/PYL ABA receptors in the presence of ABA. This interaction releases kinases that are bound to and inactivated by protein phosphatases 2C (PP2Cs), such as OST1 (open stomata 1) [136,142,143,144,145]. A similar mechanism seems to be effective for some CPKs [78,139,141], and the effects of CIPKs are also regulated by PP2Cs [140]. Some of these kinase/phosphatase pairs play critical roles in K^+^ channel regulation, as described below.

#### 3.1.1. Regulation of K^+^ Uptake and Release at the Root/Soil Interface

The first kinase to be clearly identified as a partner of a K^+^ transport system was CIPK23, interacting with the AKT1 channel in *Arabidopsis thaliana* [153,154]. Mutants in the *CIPK23* gene displayed reduced K^+^ uptake and leaf chlorosis on low-K^+^ medium [153]. CIPK23 also appears to be involved in drought resistance [185]. To activate AKT1, CIPK23 interacts with CBL1 and CBL9. Whereas *CIPK23* transcripts do not appear to display marked fluctuations in response to stress or ABA [56], with the exception of K^+^ deficiency [153], it is noticeable that the *CBL1* gene can be transiently but strongly induced by drought [159,160], and that the protein level increases in response to ABA, mannitol, and NaCl [162]. Root-specific transcript accumulation is observed in response to salt [56]. The *CBL9* gene is highly expressed in radicles of germinated seedlings; however, afterward, its expression in roots is restricted to growing zones [186]. CIPK23 phosphorylates AKT1 [153,154] and it interacts via its kinase domain with the ankyrin domain of the channel [135]. Conversely, the regulatory subunit AtKC1, closely associated with AKT1, does not interact with CIPK23 [153]. AKT1 also directly interacts with CIPK6 [135,140], whose gene expression is induced by NaCl, ABA, and mannitol, especially in roots [151], and with CIPK16 [135,140]. The CIPK6/CBL1 couple activates AKT1 in *Xenopus* oocytes in a similar way as CIPK23/CBL1 [140]. Attempts to transpose the CIPK23/CBL1 regulation to species of agronomical interest led to the characterization of the OsCIPK23/OsCBL1 complex from rice [187], which accounts for a significant part of K^+^ uptake [46], and TaCIPK23/TaCBL1 from wheat, both involved in drought tolerance [187,188]. Transcriptional responses to water-related abiotic stresses can differ between *AtCIPK23* (which seems to be insensitive to these stresses in roots and shoots) and its counterparts in other species. In whole rice seedlings, *OsCIPK23* is induced by ABA [187], and, in wheat mesophyll protoplasts, *TaCIPK23* is induced by drought, salt, and ABA [188]. It would be informative to get root-specific data. In soybean, *GmCIPK20* (closest homolog of *AtCIPK23*) is induced by drought stress in leaves and stems but not in roots [189]. Homologs of *CIPK6* were also identified in other species. *BnCIPK6* and *BnCBL1* genes (from *Brassica napus*) are both upregulated by NaCl, mannitol, and ABA in roots, in accordance with the increased salt tolerance of transgenic *Arabidopsis* plants overexpressing these genes [190].

**Figure 1:**
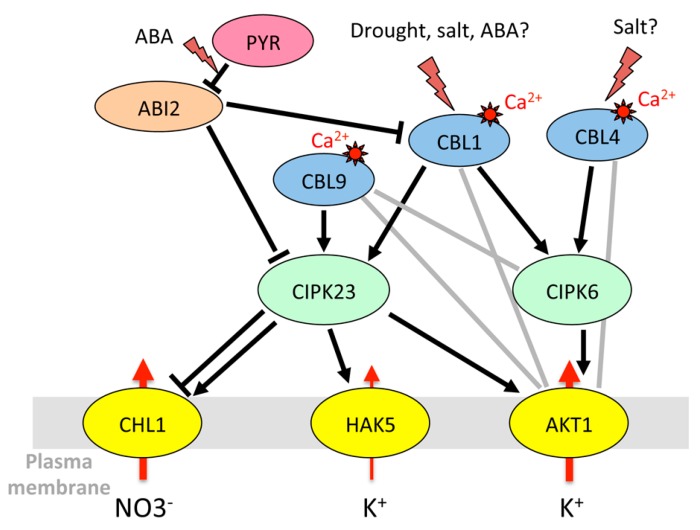
Model for cross-talks between CIPK (CBL-interacting protein kinase)/CBL (Calcineurin B-like protein) regulations of K^+^ and NO3^−^ uptake by *Arabidopsis* roots, obtained by compilations of expression and interaction data. Positive and negative regulations are depicted in black (arrows and T-bars, respectively). Indications for physical interactions (two-hybrid, bimolecular fluorescence complementation (BiFC)) in the absence of further evidence for functional effect are represented by a gray line. Red arrows symbolize the ion fluxes. The lightning symbol is used when a stress or abscisic acid (ABA) results in a change in protein amount, protein activity or protein-protein interaction. The symbol “?” stresses that the effects of the treatments on CBL1 protein accumulation [162] were only addressed in whole leaves. Also, myristoylation of CBL4 is required for its function in salt resistance [191], but was not demonstrated to occur in response to salt. References: “drought, salt, ABA?” to CBL1 [160,162]; “salt” to CBL4 [191]; ABI2 to CIPK23 and CBL4 [192]; CBL1 to CIPK6 [140,150], CBL1 and CBL9 to CIPK23 [153,154,185]; CBL4 and CBL9 to CIPK6 [150]; CIPK6 to AKT1 [135,140]; CIPK23 to AKT1 [153,154]; CBL1, CBL4 and CBL9 to AKT1 [193]; CIPK23 to HAK5 [155]; CIPK23 to CHL1 [157]. The phosphatase AtPP2CA, mainly expressed in phloem tissues [176], and not characterized for its role in root ion uptake, was not included in this figure.

Interestingly, HAK5 is the target of the same CIPK23/CBL1 complex as AKT1. CIPK23 phosphorylates HAK5, resulting in activation of the transporter, required for K^+^ uptake in low-K medium [155]. This regulation is conserved among plant species, with tomato and pepper HAK5 counterparts being activated by the *Arabidopsis* CIPK23/CBL1 complex in yeast [155]. Figure 1 summarizes the regulation of K^+^ uptake by CIPK/CBL complexes at the root/soil interface.

Recently, CPK21 was identified as a kinase activated by 14-3-3 proteins, phosphorylating the GORK C-terminal cytoplasmic domain. Following a sudden salt stress, a transient K^+^ efflux is mediated by GORK at the plasma membrane. This efflux is reduced in 14-3-3 mutants. Indeed, salt stress is expected to trigger membrane depolarization and a rise in cytosolic calcium that activates CPKs together with 14-3-3s, to promote GORK phosphorylation leading to its activation [171].

#### 3.1.2. AKT2 and Its Orthologs: Regulation of Phloem K^+^ Potential

**Figure 2:**
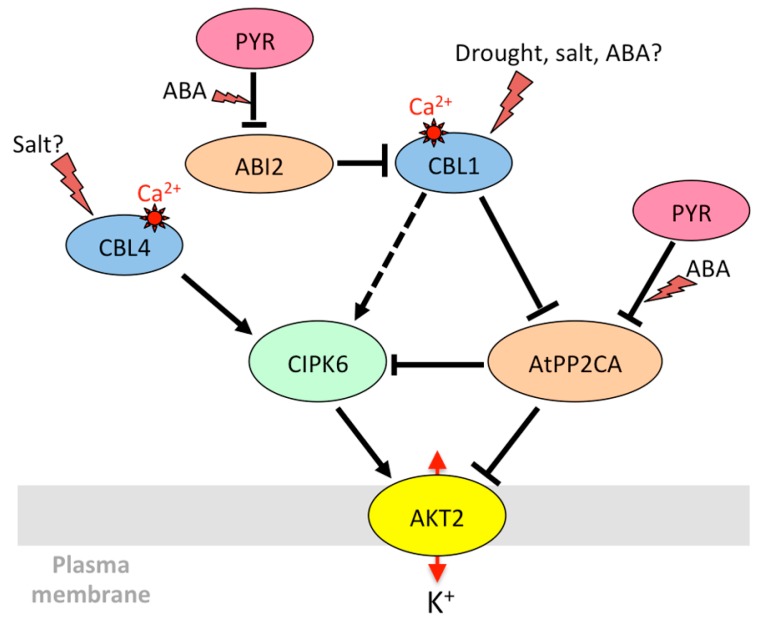
Model for the regulation of K^+^ transport through AKT2 (Arabidopsis K^+^ transporter 2) by CIPK6 and associated proteins in *A. thaliana*. See Figure 1 for details of the legend. The dotted arrow indicates that CBL1, though activating CIPK6 for AKT1 opening [131], has no functional effect on AKT2/CIPK6 [189]. References: “Drought, salt, ABA?” to CBL1 [160,162]; “Salt?” to CBL4 [191]; ABI2 to CBL1 [192]; CBL1 to CIPK6 and AtPP2CA, AtPP2CA to CIPK6 [140]; CBL4 to CIPK6, CIPK6 to AKT2 [150]; AtPP2CA to AKT2 [176].

From the beginning of its characterization, AKT2 received most of the attention due its unique gating properties, which allow it to combine a time-dependent voltage-activated current and an instantaneous ohmic current with the ability to switch between these two gating modes [31]. The mechanism via which AKT2 becomes a non-rectifying channel was deciphered progressively. The AtPP2CA protein phosphatase, which belongs to the clade A of PP2Cs involved in ABA signaling, interacts with the AKT2 channel [175,176] (Figure 2).

AtPP2CA is a negative regulator of ABA signaling [177,178] selectively interacting with some PYR/PYL/RCAR receptors [194]. Interestingly, the phosphatase changed the rectifying properties of the channel by preferentially inhibiting the leak component [176]. Later on, Michard et al. [195] revealed that phosphorylation is essential to the functioning of AKT2, by activating silenced AKT2 channels and promoting the switch to the non-rectifying, leaky mode. To date, the kinase(s) that phosphorylate AKT2 remain unidentified. Instead, the CIPK6/CBL4 couple was found to strongly activate AKT2 in a calcium-dependent but phosphorylation-independent manner, by promoting the targeting of the AKT2/CIPK/CBL complex to the plasma membrane through myristoylation of CBL4 [150]. Plants overexpressing CIPK6 are more sensitive to ABA and more tolerant to salt stress [151]. CBL4, also known as SOS3 (salt overly sensitive 3), is essential for salt resistance and potassium homeostasis [196]. Thus, regulation of AKT2 by CIPK6 and CBL4 could take part in adaptation to salt stress. A receptor-like pseudokinase, MRH1 (morphology of root hair 1), also interacts with AKT2, but has no direct functional effect. It is proposed to be involved in the recruitment of other, still unknown, partners [197].

#### 3.1.3. Stomatal Aperture Control in Case of Abiotic Stress

**Figure 3:**
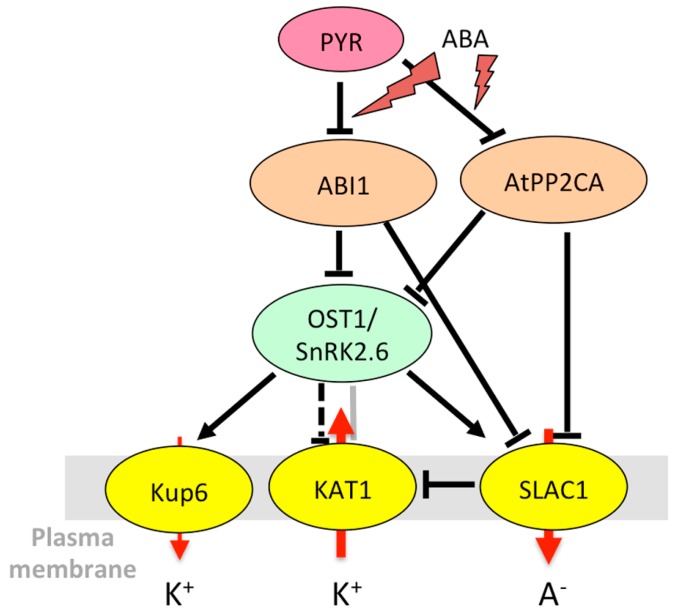
Model for the OST1 network leading to K^+^ and anion fluxes in *Arabidopsis* guard cells. The dotted T-bar indicates that the effect might be indirect. See Figure 1 for details of the legend. References: ABI1 to OST1 [144,148]; AtPP2CA to OST1 and SLAC1 [136]; ABI1 to SLAC1 [167]; OST1 to SLAC1 [136,144]; OST1 to KAT1 (in planta physiological effect) [146]; OST1 to KAT1 (interaction) [146,147]; OST1 to KUP6 [68]; SLAC1 to KAT1 [62].

During ABA-mediated stomatal closure, mechanisms leading to blue-light-induced stomatal opening are inhibited. This includes inactivation of H^+^-ATPase and Kin channels (see Reference [198] and references therein). In *A. thaliana* guard cells, the KAT1 and KAT2 channels and, probably to a lesser extent, the AKT1 channel are the major actors of K^+^ influx. Early studies revealed that the KAT1 channel was phosphorylated in vitro by 57-kD calcium-dependent and 48-kD ABA-regulated (ABR) protein kinases from *Vicia faba* [199,200]. The ABR kinase shared characteristics with a kinase named AAPK (ABA-activated protein kinase) [201], strongly suggesting that AAPK and ABR kinase are the same protein [200]. AAPK is a serine/threonine kinase specifically activated (autophosphorylated) by ABA, involved in stomatal closure [201]. Later on, the kinase OST1/SnRK2.6/SRK2E from *A. thaliana*, homologous to AAPK, was identified as a key player of ABA-mediated stomatal closure, acting upstream of ROS production [126]. The ABA-mediated inhibition of Kin channels was found reduced in the *ost1* mutants, suggesting that OST1 inhibits KAT1 [146]. OST1 phosphorylates the C-terminal cytosolic region of KAT1 [147], and direct interaction between KAT1 and OST1 can be visualized using bimolecular fluorescence complementation (BiFC) [146] (Figure 3). However, it was recently reported that KAT1 expressed in *Xenopus* oocytes is not inactivated by OST1 [62]. The mechanism of KAT1 inactivation by OST1 observed in guard cells and the effect of KAT1 phosphorylation on its K^+^ transport activity, thus, await further investigation.

**Figure 4:**
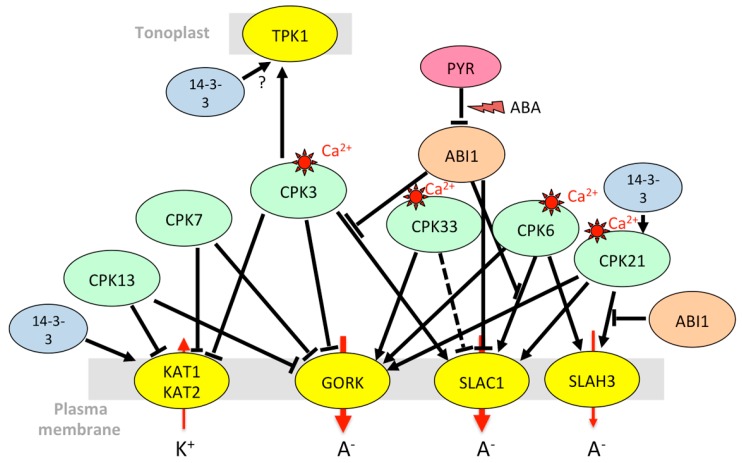
Model for integrated regulation of Shaker and anion channels by CPKs (calcium-dependent protein kinases) in *Arabidopsis* guard cells. The dotted T-bar indicates that the effect might be indirect, and the symbol “?” that the activation of TPK1 by 14-3-3 proteins was not studied in guard cells. See Figure 1 for details of the legend. References: 14-3-3 to CPK21 [171]; 14-3-3 to TPK1 [184], 14-3-3 to KAT1/KAT2 [202], ABI1 to CPK3-SLAC1 and CPK6-SLAC1 [141]; ABI1 to CPK21-SLAH3 [78]; ABI1 to SLAC1 [167]; CPK3 to TPK1 [72], CPK13 to KAT1/KAT2 [163,170], CPK13 to GORK [163]; CPK3 and CPK7 to KAT1/KAT2 and GORK [163]; CPK6 and CPK33 to GORK [163]; CPK21 to GORK [171]; CPK3 to SLAC1 [168]. CPK6 to SLAC1 [139,167,168]; CPK33 to SLAC1 [173]; CPK6 to SLAH3 [141]; CPK21 to SLAC1 [139]; CPK21 to SLAH3 [78]; CPK33 to SLAC1 [173];

Regarding calcium-dependent protein kinases (CPKs), CPK3, 4, 6, 7, 11, 13, and 33 were pinpointed as being expressed in guard cells [163]. A systematic functional screening performed in *Xenopus* oocytes led to the identification of a few of them that activate or inhibit Shaker Kin currents [163] (Figure 4). CPK13, which belongs to the small subgroup of CPKs insensitive to calcium ions, inhibits KAT2. It impairs light-induced stomatal opening but has no effect on ABA-induced stomatal closure [170], despite its inhibitory effect on GORK [163]. CPK21, which phosphorylates GORK [171], is also present in guard cells [73].

**Figure 5:**
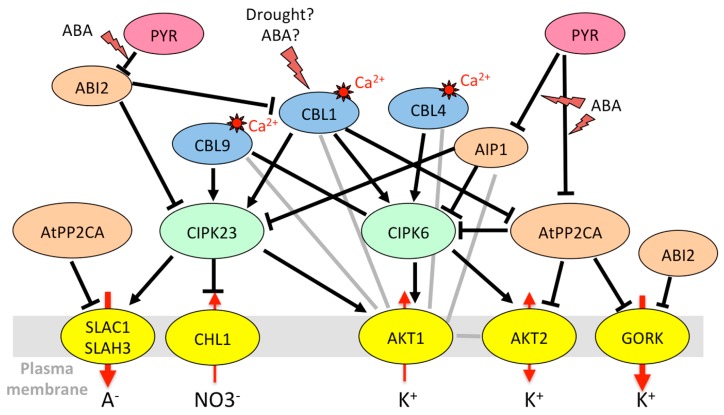
Model for the CIPK/CBL/PP2C network regulating both Shaker and anion channels in *Arabidopsis* guard cells. See Figure 1 for details of the legend. The symbol “?” indicates that the CBL1 protein, involved in stomatal movements together with CBL9 [185], is accumulated in whole leaf tissues after drought and ABA treatments [162], but this remains to be confirmed specifically in guard cells. References: “Drought, ABA?” to CBL1 [162,185]; ABI2 to CIPK23 and CBL1 [192]; AIP1 to CIPK6 and CIPK23 [140]; AtPP2CA to CIPK6 [140]; CBL1 and CBL9 to CIPK23 [153,154,185]; CBL1 to CIPK6 [140,150]; CBL4 and CBL9 to CIPK6 [150]; CBL1 to AtPP2CA [140]; CIPK6 to AKT1 [135,140]; CIPK23 to AKT1 [153,154]; AIP1 to AKT1 [135]; CIPK6 to AKT2 [150]; CBL1, CBL4 and CBL9 to AKT1 [193]; AtPP2CA to AKT2 [176]; AtPP2CA to GORK [174]; ABI2 to GORK [174]; AtPP2CA to SLAC1/SLAH3 [136]; CIPK23 to SLAC1/SLAH3 [156]; CIPK23 to CHL1 [157]; formation of AKT1-AKT2 heterotetramers [44].

AKT1 is activated by the CIPK23/CBL1 complex in guard cells as it is in roots [43] (Figure 5). It is thought to take part in the control of stomatal aperture under drought stress (but not in well-watered conditions), by limiting the extent of ABA-dependent stomatal closure. Since *akt1* plants are more resistant to drought, the exact role of AKT1 in guard cells (slowing stomatal closure for leaf cooling or maintaining photosynthesis?) remains to be clarified. Based on mutant phenotypes, it appears that its activating partners CIPK23 and CBL1/CBL9 also counteract stomatal closure in the presence of ABA and increase sensitivity to drought [185]. AKT1 also interacts with AIP1/HAI2 (AKT1-interacting protein phosphatase 1/highly ABA-induced 2) [51], an ABA-inducible clade A PP2C present in guard cells but almost absent in root epidermis and cortex [73], which itself binds CIPK23 [135,140]. AIP1, previously thought to be a positive regulator, is rather a negative regulator of the ABA signaling pathway like other clade A PP2Cs [203], interacting specifically with some PYR/PYL/RCAR receptors [204]. Together with *CIPK23*, the *CIPK6* gene, encoding another activating partner of AKT1 [140], is highly expressed in guard cells [73]. Its overexpression confers tolerance to salt stress and hypersensitivity to ABA [151]. The CIPK23 and CIPK6 kinases, thus, might have different roles in stomatal regulation. Unlike CIPK23, CIPK6 interacts with AtPP2CA, and AtPP2CA itself interacts with many CBLs including CBL1 [140]. This study also revealed the existence of many other possible channel/CIPK/CBL/PP2C complexes. A model is proposed in which AKT1, activated by a CIPK/CBL complex, is deactivated by a PP2C interacting with the kinase. Other CBLs that specifically interact with the PP2C reactivate the complex, promoting K^+^ influx [140]. It should also be noted that CBLs can directly interact with the channel and modulate its activity independent of CIPKs [59,165,193]. To our knowledge, the role of CIPK6 in guard cell movements is yet to be studied.

As the main channel mediating Kout currents at the plasma membrane of guard cells, GORK is expected to be a target of regulatory mechanisms to control stomatal closure including the closing speed. However, its regulatory network is much less characterized than that of AKT1 or KAT1. Unlike other cell types, guard cells do not induce *GORK* transcripts in response to ABA [65,73] and, therefore, need to control the channel activity via specific post-translational mechanisms. GORK activity in *Xenopus* oocytes is inhibited by CPK3, CPK7, and CPK13, and it is activated by CPK6 and CPK33 [163]. Accordingly, the *cpk33* mutants do not close their stomata in the presence of calcium. However, they are also more resistant to drought and their stomata close more efficiently in the presence of ABA [173]. GORK activity is partially inhibited by AtPP2CA [174], thus providing a fine-tuning regulation during ABA-evoked stomatal closure. Finally, the ability of GORK to change its spatial distribution at the guard-cell plasma membrane, at least in response to high K^+^, suggests the existence of additional post-translational regulatory mechanisms [205]. KUP6, also mediating K^+^ efflux from guard cells, is phosphorylated by OST1 [68], while this kinase has no effect on GORK activity in *Xenopus* oocytes [174].

Although much less studied than K^+^ fluxes at the plasma membrane, K^+^ loading and unloading of vacuoles play a central role in the control of guard-cell turgor. The TPK1 tonoplast channel seems to be the main K^+^-selective channel involved in that process. It is phosphorylated by CPK3 that is highly expressed in guard cells [73] and responds to calcium elevation in the cytosol [72]. In response to ABA, TPK1 is also phosphorylated by a receptor-like kinase, named KIN7 (kinase 7), resulting in channel activation and stomatal closure [119].

#### 3.1.4. Regulation of K^+^ Transport in Grape Berry by CIPK/CBL Complexes and SnRK2s

The AKT1-related VvK1.1, VvK1.2, and AKT2-related VvK3.1 genes are strongly upregulated by drought stress (VvK1.1 also by ABA treatment), and these channels are activated by CIPK/CBL couples [50,51,59]. Grapevine CIPK and CBL genes display differential responses to various abiotic stresses [206]. The *VvCIPK04* gene, encoding a kinase strongly activating VvK1.2, is upregulated by drought stress in the ripening berry [51]. In the ABA signaling cascade, SnRK2 kinases and clade A PP2Cs, which are likely to modulate the activities of Shaker K^+^ channels, interact with each other in a specific manner and corresponding genes are differentially regulated [207]. Among the kinases, OST1 counterparts SnRK2.1 and SnRK2.4 display a positive signal in two-hybrid tests with the Shaker channel VvK2.1, the ortholog of KAT1 [207]. In the grape berry, ABA induces berry ripening [208] and ABA content increases under heat [209,210]. It is, therefore, likely that the increase in K^+^ berry content correlated to temperature increases can be attributed at least in part to Shaker channels in relation to SnRK2 and CIPK kinases.

#### 3.1.5. Vacuolar K^+^ Uptake and Release in Response to Salt Stress

As highlighted above, maintaining K^+^ homeostasis is crucial. In the case of salt stress, Na^+^ is accumulated in vacuoles, and K^+^ is released from the vacuole to maintain the cytosolic K/Na ratio. For efficient sequestration of Na^+^ in the vacuoles, slow and fast vacuolar currents allowing Na^+^ leakage from the vacuole to the cytosol must be inactivated [211]. Under salt stress, they are suppressed by polyamines and an increase in vacuolar calcium. Conversely, TPK1 remains active. It mediates K^+^ flux back to the cytosol to counterbalance Na^+^ entry into the vacuole and compensates for K^+^ efflux through GORK at the plasma membrane [211]. In poplar, overexpression of *PeTPK1* results in improvement of salt tolerance [212]. Leaf extracts from *Arabidopsis* salt-stressed plants, but not those of control plants, in vitro phosphorylate the TPK1 N-terminus [72]. Looking for kinases that would phosphorylate TPK1, Latz et al. [72] identified several CPKs including CPK29, induced by salt stress at the transcriptional level, and CPK3, known to be involved in stress responses. CPK3 responds to cytosolic calcium elevation triggered by salt stress, and phosphorylates a serine residue in the N-terminus of TPK1 [72] (Figure 4). Phosphorylation at this site enables GRF6 (growth-regulating factor 6), a 14-3-3 protein, to bind to TPK1 and activate the channel [184].

### 3.2. Regulation by Vesicle-Associated Proteins

As discussed above for AKT2, an increase in channel activity at the plasma membrane can result from a change in channel gating properties (e.g., due to phosphorylation) or from a regulation of membrane targeting. Screening for guard-cell tobacco RNAs able to induce ABA-dependent Cl^−^ efflux in *Xenopus* oocytes, Leyman et al. [179] isolated Nt-SYR1 (*Nicotiana tabacum* syntaxin-related protein), a syntaxin (Qa SNARE, soluble N-ethylmaleimide sensitive factor attachment receptor) associated with the plasma membrane. This protein accumulates in leaves after ABA treatment [180]. A cleavage fragment of this syntaxin (Sp2) not only blocked ABA-dependent regulation of chloride channels, but also that of inward and outward K^+^ channels [179]. Visualization of KAT1 fused to GFP revealed that SP2 and SYP121 (*Arabidopsis* homolog of Nt-SYR1) modify KAT1 distribution and mobility at the plasma membrane [213]. During stomatal closure (or after ABA treatment [214]), KAT1 is internalized, and SYP121 favors KAT1 recycling to the plasma membrane for stomatal reopening [182]. SYP121 also binds to AtKC1, and this interaction leads to the activation of the AKT1/AtKC1 complex [181]. The binding site of SYP121 to K^+^ channels is the voltage sensor, and binding is favored by hyperpolarization, thereby allowing coordination of vesicle trafficking for cell volume adjustment and K^+^ channel voltage-dependent activation [158]. Other SNAREs associated with trafficking vesicles, which belong to the VAMP (vesicle-associated membrane protein) family (R-SNAREs), also interact with KAT1 and AtKC1. Contrary to SYP121, VAMP721 suppresses the KAT1 and AKT1/AtKC1 currents in *Xenopus* oocytes. Altogether, SYP121 and VAMP have complementary actions on K^+^ channel activities [183] (Table 2).

## 4. Cross-Talks with Anion Transport

As the major cation in plant cells, K^+^ has to be neutralized by organic acids and mineral anions. To maintain electro-neutrality, membrane K^+^ transport is accompanied by the transport of anions. In guard cells, efflux of K^+^ is driven by an efflux of anions. From the beginning of the characterization of channel regulatory proteins, it is striking that elements of the calcium and ABA signaling pathways (PP2C and their associated kinases) can be involved in the regulation of both K^+^ and mineral anion transport. The main mineral anions in plants are Cl^−^ and NO3^−^. Anion channels of the SLAC1/SLAH (slow anion channel 1/ SLAC1 homolog) family (five members in *Arabidopsis*) mediate voltage-independent slow and massive chloride and nitrate fluxes at the plasma membrane, for root anion uptake, transfer of anions to the shoot, and stomatal closure [215]. Recent findings indicate that the anion channels SLAC1 and SLAH3 interact with Shaker channels. KAT1 (but not its closest homolog KAT2) is inhibited by both SLAC1 and SLAH3 via direct physical interaction [62].

In guard cells, SLAC1 and SLAH3 mediate nitrate and chloride transport. SLAC1 was found inhibited by AtPP2CA [136], like AKT2 [176] and GORK [174]. This mechanism would permit a fine coordination of K^+^ and anion effluxes and modulation of the speed of ABA-triggered stomatal closure. SLAC1 interacts with and is activated by the OST1 kinase, whose activity is inhibited by AtPP2CA and its close relative ABI1 (ABA-insensitive 1) in an ABA-dependent manner [136,144].

Like OST1, kinases of the CPK family, activated by calcium, also interact with SLAC1 and SLAH3 to modulate anion efflux in guard cells. CPK21 and CPK23 both activate SLAC1 and SLAH3 in an ABA-and ABI1-dependent manner, but with contrasting effects on channel currents and calcium sensitivity [78,139,141]. In the *cpk3 cpk6* double knock-out mutant, Ca^2+^- and ABA-activated S-type anion channel currents are abolished, and stomatal closure is partially impaired [166]. Accordingly, CPK6 was found to activate SLAC1 in *Xenopus* oocytes and to phosphorylate the SLAC1 N-terminus at a much higher speed than CPK23 and OST1 [167]. CPK21 [139] and CPK3 [141] also activate SLAC1 in oocytes, but only in their truncated, constitutively active form, suggesting that they are weakly sensitive to calcium and operate only in response to an increase in cytosolic calcium. Contrary to CPK3, CPK6, CPK21 and CPK23, which activate SLAC1 and SLAH3, CPK33 counteracts ABA-dependent slow anion channel activation in guard-cell protoplasts [173]. Finally, it is interesting to note that the CIPK23/CBL1 complex, already known to regulate the K^+^ channel AKT1 and the high-affinity transporter HAK5, also interacts with and activates SLAC1 and SLAH3. Like CPK23, it phosphorylates the N-terminus of SLAC1 in a calcium-dependent manner, but there exist specificities for target phosphorylation sites [156].

In the nitrate transporter family, CHL1 (NRT1.1/NPF6.3) (chlorate-resistant 1, nitrate transporter 1, NRT1/PTR family 6.3), involved in root NO3^−^ uptake [80], is also targeted by the same kinases and phosphatases. Under low-nitrate conditions, CIPK23/CBL9 phosphorylates CHL1, and converts it into a high-affinity nitrate transporter. However, when nitrate level is high, the transporter returns to the low-affinity state and the CIPK/CBL complex inhibits nitrate transport through CHL1 [157]. CBL1 also associates with CIPK23 to decrease NO3^−^ uptake under high external nitrate in *Xenopus* oocytes [192]. This inhibitory effect is reversed by the clade A PP2Cs ABI1 and ABI2 [192]. In addition to its role in NO3^−^ uptake, CHL1 is also involved in stomatal opening and plant drought susceptibility [81]. CIPK23 and CBL1 are both expressed in guard cells [73]. Since the CBL1 protein level is enhanced in response to ABA or osmotic stress (Table 2), the inhibition of CHL1 by the CIPK23/CBL1 complex could be a means to control anion influx that would counteract stomatal closure.

Clade A PP2C phosphatases are at the center of ABA signaling pathways. Their incorporation into kinase/channel complexes most often results in complete abolition of the effect of the kinases. Indeed, AtPP2CA, ABI1, and/or ABI2 drastically inhibit the effect of OST1 [136,144,167], CPK23 [139], CPK6 [167], CPK21 [78], and CIPK23 [156] on SLAC1 or SLAH3 in *Xenopus* oocytes. AtPP2CA [136,138] and ABI1 [138,144] directly inactivate OST1, but not CPK6 [168], despite the existence of an ABI1–CPK6 interaction [141]. In parallel, AtPP2CA directly inhibits AKT2 [176], GORK [174], and SLAC1 [136] activities, and ABI1 inhibits SLAC1 [167]. ABI1 and ABI2 also suppress the inhibitory effect of CIPK23/CBL1 on CHL1-mediated nitrate uptake [192]. Thus, PP2C phosphatases act at several levels on channel activities: inhibition of upstream kinases [78,136,144] and/or direct inactivation of channels [136,174] via binding [174] or dephosphorylation [167,168].

In summary, common regulatory pathways can control the transport of cations and anions through diverse transport systems to maintain ion homeostasis. Kinases play a central role in these regulations. For instance, OST1 causes stomatal closure both by leading to the inactivation of Kin channels [146] and by activating anion efflux through SLAC1 [33,136]. It also phosphorylates AtRBOHF (respiratory burst oxidase homolog plasma membrane NADPH oxidase) that produces ROS for calcium signaling, in accordance with its role in stomatal closure [42]. However, things can be more complex since some regulations seem to go in opposite directions. A big node in the regulatory network is the kinase CIPK23 that interacts with and phosphorylates multiple membrane targets including AKT1, HAK5, SLAC1, and CHL1 [127]. It activates both AKT1, which prevents stomatal closing, and SLAC1/SLAH3 channels, which are involved in stomatal closure. The phenotype of the *cipk23* mutants (enhanced tolerance to drought stress [43]) rather fits with its role in AKT1 regulation. In the CPK family, CPK13 inhibits Kin channels KAT1 and KAT2 [170], but also the K^+^ efflux channel GORK [163]. CPK33 activates GORK and favors calcium-dependent stomatal closure [163] but downregulates slow anion channels and slows down ABA-mediated stomatal closure [173]. The *cpk21* mutant also displays resistance to mannitol [172], while CPK21 activates SLAH3 [172], which would result in enhanced stomatal closure and drought resistance. A multiplicity of targets (Figure 1, Figure 2, Figure 3, Figure 4 and Figure 5) and variations in spatiotemporal gene expression might explain why mutant phenotypes are not always consistent with what is expected when considering only one mechanism. Furthermore, knocking down one gene can result in the deregulation of other genes in stress signaling processes, as shown for CPK23 [139] and CPK21 [172]. Clade A PP2Cs such as AtPP2CA, ABI1, and ABI2 are also key nodes in ABA and calcium-dependent signaling. They target multiple proteins in the cytosol and nucleus to allow plant adaptation to multiple stimuli [216]. Depending on environmental conditions, they trap or release kinases that modulate the activity of ion channels and transporters, and they also directly bind to ion membrane transporters and CBL proteins. Among CBLs, CBL1 is a big hub. The gene is upregulated by salt stress, while ABA and abiotic stresses (salt, osmotic) result in the accumulation of the protein. The protein is under control of PP2Cs, and it has multiple downstream targets that can interact with each other (Figure 1, Figure 2 and Figure 5). This shows the inter-connection of different signaling pathways associating ion transporters (cations, anions), kinases (CIPKs, CPKs, SnRK2), CBLs, and PP2C phosphatases, and suggests the existence of multi-protein complexes that can be recruited according to the plant’s status and environment.

## Figures and Tables

**Table 1:** Plant K^+^ transport systems described in this study and their regulations in response to ionic and osmotic stresses.

Gene Name ^1^	ID	Protein Family	Function	Type of Stress	Transcriptional Regulation (Drought, ABA, Heat Stress, NaCl)	Protein Level/Activity
*AKT1*	At2g26650	Shaker	K^+^ uptake by roots [42], inhibition of stomatal closure in response to drought [43]	ABA	No significant change in roots, downregulation in shoots [44] and seedlings [45]	
				NaCl	No significant change in roots and shoots [44]	Inhibition of currents by intracellular Na^+^ [43]
*OsAKT1*	LOC4326245	Shaker	K^+^ uptake by roots [46,47]	NaCl	Downregulation [48], downregulation in the exodermis in salt-tolerant lines [49]	
*VvK1.1*	VIT_11s0016 g04750	Shaker	K^+^ uptake in roots and phloem [50]	Drought	Induction in leaves and berries, downregulation in roots [50]	
				ABA	Induction in leaves, not in roots [50]	
*VvK1.2*	VIT_04s0008 g04990	Shaker	K^+^ uptake by flesh cells [51]	Drought	Strong induction in berries from véraison [51]	
*AKT2*	At4g22200	Shaker	Regulation of phloem membrane potential [52,53]	ABA	Induction in leaves [44,54], not in guard cells [55]	
				Mannitol, NaCl, drought	Moderate induction in leaves [56]	
				NaCl	Induction [57]	
				Heat	Induction [58]	
*VvK3.1*	VIT_12s0034 g02240	Shaker	Phloem transport, leaf movements [59]	Drought	Induction in leaves and (moderately) in berries, not in roots [59]	
*KAT1*	At5g46240	Shaker	Stomatal opening [60,61]	ABA	Down-regulation in guard cells [55]	
				Drought	No change in guard cells [62]	
				NaCl	No significant change [44]	
*KAT2*	At4g18290	Shaker	Stomatal opening [60,61]	ABA	Down-regulation in guard cells [55]	
*AtKC1*	At4g32650	Shaker	K^+^ uptake in root hairs together with AKT1 [35]	ABA	Transient down-regulation [44]	
				NaCl	High induction in leaves, not in roots [44]	
*SKOR*	At3g02850	Shaker	K^+^ loading of the xylem in roots [63]	ABA	Downregulation [63]	
				NaCl	No significant change [44], strong induction [57]	
*GORK*	At5g37500	Shaker	Stomatal closure [64]	ABA	Induction in seedlings, cultured cells, root hair protoplasts, not in guard cells [65]	
				Heat	Induction in roots [56]	
				Water stress	Induction in leaves [65]	
				NaCl	Induction in roots [56]	
*HAK5*	At4g13420	KUP	High-affinity K^+^ uptake from the soil [66]	NaCl	Strong downregulation [67]	
				NaCl, drought, mannitol	Unclear [56]	
*KUP6*	At1g70300	KUP	K^+^ efflux from roots and stomata, ABA- and auxin-dependent inhibition of lateral root formation [68]	NaCl	Induction [57]	
				Heat	Induction (Genevestigator, https://genevestigator.com, [58])	
				Mannitol	Strong induction [56]	
*KUP8*	At5g14880	KUP	K^+^ efflux from roots and stomata, ABA- and auxin-dependent inhibition of lateral root formation [68]	ABA, NaCl, mannitol	Slight downregulation [56], especially in roots (NaCl, mannitol)	
*OsHAK1*	LOC4335729	KUP	K^+^ acquisition [69]	Water stress	Transient induction in roots and shoots [69]	
*OsHAK5*	LOC4326945	KUP	K^+^ acquisition, root to shoot transport [70]	NaCl	Induction in roots and shoots [70]	
*OsHAK21*	LOC9269115	KUP	K^+^ uptake and distribution [71]	NaCl	Induction in roots and shoots [71]	
*TPK1*	At1g02880	Two-pore channels	K^+^ homeostasis, guard cell vacuolar K^+^ release [41]	NaCl	No significant change [72]	Induction of protein phosphorylation [72]
				ABA	No change [45,73]	
*SLAC1*	At1g12480	C4 dicarboxylate transporters	Stomatal closure [74,75]	Drought	Short treatment: no change in roots and shoots [66], strong induction in guard cell protoplasts after a few days [62]	
				ABA	No change [45,73,76]	
				NaCl	Moderate induction in shoots [56]	
				Mannitol	No significant change in roots and shoots [56]	
*SLAH3*	At5g24030	C4 dicarboxylate transporters	Cl^−^ root to shoot translocation [77], NO_3_^−^-mediated stomatal closure [78], pollen tube growth [79]	Drought	Short treatment: downregulation in roots [56]; strong induction in guard cell protoplasts after a few days [62]	
				ABA	No change [45]	
				NaCl	Downregulation in roots [56,77]	
				Mannitol	Downregulation in roots [56]	
				PEG	Strong downregulation in roots [77]	
*CHL1* /*NRT1.1* /*NPF6.3*	At1g12110	NPF	Nitrate uptake [80], stomatal opening [81], nitrate sensing, auxin transport [82]	ABA	Unclear, depends on tissue/experimental conditions [45]	
				NaCl, mannitol	Unclear, transient down-regulation in roots? [56]	

^1^ Gene names: AKT: *Arabidopsis* K^+^ transporter, KAT: K^+^ *Arabidopsis* transporter, AtKC: *Arabidopsis thaliana* K^+^ channel, SKOR: stelar K^+^ outward rectifyer, GORK: guard cell outward rectifying K^+^ channel, HAK: high affinity K^+^ transporter, KUP: K^+^ uptake permease, TPK: two-pore (or tandem pore) K^+^ channel, SLAC: slow anion channel, SLAH: SLAC1 Homologue, CHL: chlorate-resistant, NRT: Nitrate Transporter, NPF NRT1/PTR Family. Genes originate from *Arabidopsis thaliana* unless otherwise stated. *Os*: *Oryza sativa*, *Vv*: *Vitis vinifera*.

**Table 2:** Regulatory partners of plant K^+^ transport systems, their targets among K^+^ and mineral anion transport systems, and their regulations in response to ionic and osmotic stresses.

Gene Name ^1^	ID	Protein Family	Plasma Membrane Targets	Type of Stress	Transcriptional Regulation (Drought, ABA, Heat Stress, NaCl)	Protein Level/Activity
*OST1/* *SnRK2.6/* *SRK2E*	At4g33950	SnRK2 kinases	KAT1 [146,147], SLAC1 [136,144], KUP6 [68]	ABA	No change in transcript level in whole plants [126], induction in guard cells [55]	Induction of kinase activity in roots and guard cell protoplasts [126]
				Low air humidity		Induction of kinase activity in leaves [148]
				NaCl, sorbitol		Induction of kinase activity in roots [148]
				NaCl	Slow induction [56]	
				Drought	Moderate induction [149]	
*CIPK6*	At4g30960	SnRK3 kinases	AKT1 [135,140], AKT2 [150]	ABA, mannitol	Induction in seedlings (including roots) [151]	
				NaCl	Induction in shoots [56] and in seedlings (including roots) [151]	
*CIPK16*	At2g25090	SnRK3 kinases	AKT1 [135]	NaCl	Induction in root stele [152]	
*CIPK23*	At1g30270	SnRK3 kinases	AKT1 [135,153,154], HAK5 [155], SLAC1 [156], CHL1 [157]	ABA, drought, mannitol, NaCl	No change [45]	
*CBL1*	At4g17615	CBL	AKT1 [158]	Drought	Short air treatment: transient induction in roots only [56]; strong and transient induction after 1-3h [159,160]; long-term induction in soil [149]	
				ABA	No significant change in mesophyll and guard cells [73] and in seedlings [160,161]	Protein accumulation in leaves [162]
				NaCl	Transient induction in roots [163]	Protein accumulation in leaves [162]
*CBL4/* *SOS3*	At5g24270	CBL	AKT1 [158]	NaCl, mannitol	Induction in roots [56], no significant change in seedlings [164]	
				ABA	Down-regulation in guard cells [73]	
*CBL9*	At5g47100	CBL	AKT1 [158]	ABA, NaCl, mannitol, drought	No significant change [45]	
*CBL10*	At4g33000	CBL	AKT1 [165]	Mannitol	Down-regulation in shoots [56]	
				NaCl	Transient and moderate induction [72]	
*CPK3/* *CDPK6*	At4g23650	CPK/CDPK	KAT1/KAT2, GORK [163], TPK1 [72]	NaCl	No change in seedlings [72]	
				ABA	Slight down-regulation in guard cells [73,76]	
*CPK6/* *CDPK3*	At2g17290	CPK/CDPK	GORK [163], SLAC1 [139,166,167,168]	ABA, mannitol, NaCl	No change [45,73]	
				NaCl, PEG	Transient induction [169]	
*CPK13*	At3g51850	CPK/CDPK	KAT1/KAT2 [170], GORK [163]	NaCl, drought	No significant change [56]	
				Mannitol	Downregulation [56]	
				ABA	No change in guard cells [73,76]	
*CPK21*	At4g04720	CPK/CDPK	GORK [171], SLAC1 [139], SLAH3 [78]	ABA, drought, mannitol, NaCl	No significant change, except perhaps a slight down-regulation in response to ABA in guard cells [45]	
				Mannitol		Enhancement of kinase activity [172]
*CPK33*	At1g50700	CPK/CDPK	GORK [163], slow anion channels? [173]	ABA, drought	Moderate up-regulation [173]	
*ABI1*	At4g26080	Clade A PP2C phosphatases	SLAC1 [167]	ABA	Induction in guard cells [55,73,76] and seedlings [45]	
				NaCl, mannitol	Induction in roots and shoots [56]	
				Heat	Twofold induction in leaves [58]	
*ABI2*	At5g57050	Clade A PP2Cs	GORK [174]	ABA	Induction in guard cells [55,73,76] and seedlings [45]	
				NaCl, mannitol	Induction in roots and shoots [56]	
*AtPP2CA*	At3g11410	Clade A PP2Cs	AKT2 [175,176], GORK [174], SLAC1 [136]	ABA	Strong induction in guard cells [55,73,76] and whole plants [56,176,177,178]	
				NaCl, mannitol	Induction in roots and shoots [56]	
				Heat	Fourfold induction in leaves [58]	
*AIP1*	At1g07430	Clade A PP2Cs	AKT1 [135,140]	ABA	Induction in seedlings [56] and guard cells [55]	
				NaCl, mannitol	Strong induction in roots (NaCl) or shoots (mannitol) after 3 hours [56]	
				Heat	Strong induction in leaves [58]	
*NtSYR1*	LOC107768839	Syntaxins	*Nicotiana* K and Cl channels [179]	NaCl	Induction of transcripts in leaves [180]	Protein accumulation in leaves [180]
				ABA	Transient induction of transcript in leaves [179]	Protein accumulation in leaves, but not in roots [180]
*SYP121*	At3g11820	Syntaxins	AtKC1, AKT1-AtKC1 [181], KAT1 [182]	NaCl	Induction in roots, not in leaves [56]	
				ABA	No induction in seedlings and guard cells [45]	
*VAMP721*	At1g04750	Synaptobrevin-like	KAT1, AtKC1 [183]	ABA, NaCl, drought, mannitol	No significant change [45]	
*VAMP722*	At2g33120	Synaptobrevin-like	KAT1, AtKC1 [183]	ABA, NaCl, drought, mannitol	No significant change [45]	
*GRF6*	At2g06200	14-3-3	TPK1 [184]		Low expression	

^1^ Gene names: OST: open stomata, CIPK: CBL-interacting protein kinase, SnRK/SRK: SNF1-related protein kinase, CBL: calcineurin B-like, SOS: salt overly sensitive, CPK or CDPK: calcium dependent protein kinase, ABI: ABA-insensitive, PP2C: protein phosphatase 2C, AIP: AKT1-interacting protein, SYR: syntaxin-related protein, SYP: syntaxin-related protein, VAMP: vesicle-associated membrane protein. All genes originate from *Arabidopsis thaliana* except *NtSYR1* (from *Nicotiana tabacum*).
